# Psychosocial interventions for military personnel with PTSD: protocol of a systematic review and network meta-analysis

**DOI:** 10.1136/bmjopen-2026-124637

**Published:** 2026-10-05

**Authors:** Ioannis Angelakis, Sarah Donegan, Karen Hughes, Laura Goodwin, Ffion Curtis, Alison Bryant, David Sparks, Michelle Maden, Anum Nisar, Thomas Spain, Ahmed Waqas, Catrin Tudur-Smith

**Affiliations:** 1 Department of Primary Care & Mental Health, University of Liverpool, Liverpool, UK; 2 Cambridgeshire and Peterborough Mental Health Partnership NHS Trust, Fulbourn, UK; 3 Department of Clinical Psychology and Psychological Therapies, Norwich Medical School, Faculty of Medicine and Health Sciences, University of East Anglia, Norwich, UK; 4 Department of Health Data Science, Institute of Population Health, University of Liverpool, Liverpool, UK; 5 Policy and International Health, World Health Organization Collaborating Centre on Investment for Health and Well-being, Public Health Wales, Wrexham, UK; 6 Public Health Collaborating Unit, School of Health Sciences, Bangor University, Bangor, UK; 7 Division of Health Research, Lancaster University, Lancaster, UK; 8 Liverpool Reviews and Implementation Group (LRiG), University of Liverpool, Liverpool, UK; 9 Patient and Public Involvement Contributor, University of Liverpool, Liverpool, UK

**Keywords:** Stress Disorders, Traumatic, Acute, Network Meta-Analysis, Particulate Matter

## Abstract

**Abstract:**

**Introduction:**

Post-traumatic stress disorder (PTSD) is a serious mental health condition. It may develop following exposure to an extremely threatening event. Military personnel face an increased likelihood of developing PTSD because they sometimes bring experiences of difficult events from before they joined the military and because they have higher odds of being exposed to potentially traumatic events during service. To support the mental health of military personnel, several psychological interventions have been developed to help them manage PTSD symptoms. Although there is a lot of research demonstrating how well these interventions work, we do not know how the different interventions (eg, trauma-informed therapy, cognitive-behavioural therapy) compare. Because many of these interventions include more than one type of treatment (eg, behavioural activation), we do not know which specific component works best.

**Methods and analysis:**

To explore these questions, we will identify previous studies that have examined interventions for PTSD in military personnel. We will then use network meta-analysis methods to make comparisons between all interventions, including some that have never been compared against each other in previous studies, to identify the best treatments. We also aim to identify which individual components are effective, even when delivered in combination. We will work with people with lived experiences, researchers, and mental health professionals to guide the project stages. Overall, this project aims to improve outcomes for military and ex-military personnel with PTSD by (1) identifying effective treatments, (2) establishing the relevance and transferability of the findings to a wider audience and (3) offering suggestions that will have immediate implications for clinical practice.

**Ethics and dissemination:**

No ethics approval is required as this study only involves a systematic review of published literature. The findings will be published in peer-reviewed journals and presented at relevant conferences.

**PROSPERO registration number:**

CRD420251081519.

**STRENGTHS AND LIMITATIONS OF THIS STUDY**

- Network meta-analysis will synthesise direct and indirect evidence, subject to the assumptions of transitivity and consistency.
- Component network meta-analysis will model interventions according to their constituent components, although estimates may be limited by sparse data and assumptions regarding component effects and interactions.
- The review will include input from people with lived experience, clinicians and researchers to enhance the relevance, interpretability and potential clinical applicability of the findings.

## Introduction

 This project aims to gather the evidence needed to develop better approaches for managing the symptoms characterising post-traumatic stress disorder (PTSD) in both serving and ex-serving military personnel (hereafter referred to as military personnel, unless a specific group is mentioned). PTSD may occur in any person who has witnessed or experienced a traumatic, life-threatening or horrible event, or series of adverse events and/or circumstances. People who have developed PTSD may reexperience traumatic events, experience distressful thinking, feelings or reminders of the traumatic event, and have poor mood and elevated arousal.^1^ Although PTSD can affect anyone, with about 6% of the general population reporting symptoms, it is particularly common in military personnel, with rates of 7% and 11% found among those who are currently serving or who had previously served in the past, respectively.^2 3^ These rates can rise even further, reaching 13% and 18% respectively among those deployed in combat.^2^ As of April 2024, the full-time UK armed forces included about 148 230 people, with more than half serving in the Army,^4^ which makes these figures even more significant.

Various PTSD treatments for military personnel have been established, including trauma-focused cognitive-behavioural therapy (CBT),^5^ cognitive processing therapy,^6^ prolonged exposure^7^ and eye movement desensitisation and reprocessing (EMDR).^8^ Their effectiveness has been established via reviews and meta-analyses.^9–13^ A recent network meta-analysis^14^ focusing on trauma-focused interventions and studies published until 2019 found that such psychological treatments could be effective in managing PTSD among individuals with complex trauma histories. However, this paper did not focus exclusively on military personnel and included other populations such as survivors of childhood sexual abuse, refugees or those affected by domestic violence and war. Notably, it excluded studies focusing on serving military personnel. However, in the 15 trials which involved ex-serving military personnel only, psychological interventions were found to reduce PTSD symptoms. It is worth noting that this effect size was smaller compared with those for other populations and benefits were not maintained at 6-month follow-up.^14^

Although military personnel share some experiences with other high-stress professions, there is strong evidence that the unique factors associated with the military may also influence the development of PTSD in this group.^15 16^ These factors may include experiences of childhood trauma, especially among military personnel seeking treatment for PTSD.^17 18^ These adverse childhood experiences may not only increase the likelihood of developing symptoms of PTSD but can often complicate treatment outcomes. This may be attributed to the fact that such past childhood events may interact with service-related traumas creating a complex mental health profile. Other military stressors may include prolonged exposure to combat, witnessing violence, a lack of social support structure and moral injury from actions and/or decisions under pressure.^19^ Overall, the extant literature has shown that a history of premilitary trauma can elevate the risk of PTSD.^17–19^ It can also increase the risk of developing other major mental health problems, such as depression, and contribute to reduced health-related quality of life, especially in those with multiple types of trauma.^17–19^

Lifetime exposure to trauma has been identified as a predictor of PTSD among new recruits.^20^ The interplay between preservice and service-related trauma may increase risk further.^15^ Stigma associated with seeking mental health support within the military culture can also delay treatment and result in symptom worsening. These findings highlight the need for a new network meta-analysis that focuses exclusively on military populations to provide evidence for optimising PTSD treatment in these vulnerable populations.

The aim of this review and component network meta-analysis (component-NMA) is to evaluate existing clinical research on psychological interventions for PTSD among military personnel. Our specific objectives are: Objective 1: To evaluate the relative effectiveness of psychological interventions, such as CBT, cognitive processing therapy, and Prolonged Exposure, in alleviating symptoms, reducing adverse effects and improving health-related quality of life in military personnel with PTSD. Objective 2: To estimate the comparative effectiveness of individual components, such as behavioural activation, cognitive restructuring, or mindfulness, in psychological interventions aimed at improving symptoms, reducing adverse events, and enhancing health-related quality of life for military personnel with PTSD and Objective 3: To compare the effectiveness of psychological interventions across subgroups defined by age, gender, ethnicity, deprivation/socioeconomic status, educational level, experience of previous trauma and other protected characteristics, as identified under the FOR EQUITY and PROGRESS-Plus frameworks.^21^ Furthermore, this objective will include an assessment of where such sociodemographic data have not been recorded in studies, to highlight potential gaps in equity, diversity and inclusion reporting and inform future research priorities.

## Methods

This protocol has been developed with reference to the Preferred Reporting Items for Systematic Reviews and Meta-Analyses (PRISMA) extension for protocols and the final systematic review will be reported according to the PRISMA extension for Network Meta-analysis.^22 23^ The protocol has been registered on PROSPERO (number CRD420251081519). The protocol was developed in consultation with an advisory group comprising eight experts, including clinical, methodological and public advisors, to ensure relevance, methodological rigour and applicability to stakeholder needs. This systematic review will be conducted from 1 January 2026 to 1 June 2027.

### Eligibility criteria

Study design: Individual randomised and cluster-randomised controlled trials (RCTs) will be included. Exclusions include non-RCTs, theses, dissertations and conference abstracts.

Population: Serving or ex-serving military personnel aged 16 or older with PTSD, diagnosed either formally through clinical assessments or informally using validated self-report scales.

Intervention: Psychological and psychosocial interventions aimed at treating PTSD, alone or in combination as multicomponent interventions. Interventions include but are not limited to, psychological interventions such as CBT, cognitive or emotional processing therapies, and mindfulness or meditation; and psychosocial interventions including peer support and psychosocial and vocational rehabilitation. They may be delivered in various formats, including face-to-face sessions, group settings, digital platforms or self-help resources.

Comparator: Comparators may be psychological and psychosocial interventions or no intervention, usual care and waitlist controls.

### Outcomes

The primary outcome will be PTSD symptom severity post-treatment, measured with validated scales such as the Clinician-Administered PTSD Scale, the PTSD Checklist, the Impact of Event Scale–Revised, the International Trauma Questionnaire, the PTSD Symptom Scale, the Brief Symptom Inventory, the Symptom Checklist-90 or the Global Assessment of Functioning.

Secondary outcomes include

- PTSD symptom severity at long term follow-up.
- Depression.
- Anxiety.
- Dissociation.
- Functional somatic syndromes.
- Suicidal ideation.
- Suicidal attempts.
- Suicidal completions.
- Health-related quality of life.

Where multiple measures are reported for the same outcome within a study, we will select one measure based on clinical relevance and consistency across studies. Where necessary, sensitivity analyses will be conducted to assess the impact of outcome selection.

### Search strategy

We will search multiple databases from inception, including MEDLINE, CINAHL, Embase, PsycINFO, Web of Science, Cochrane CENTRAL, the International Clinical Trials Registry Platform, ClinicalTrials.gov, the CEA Registry, the International Health Technology Assessment database and the NICE website. An experienced information professional (MM) developed an initial exploratory search in MEDLINE using both subject headings and free-text searches, combined with advanced techniques such as truncation, proximity operators, and phrase frequency analyses to ensure sensitivity. This initial scoping search allowed us to develop a PubMed strategy (online supplemental table 1). The keywords were also informed by previous reviews and input from our Patient and Public Involvement (PPI) group. The search will then be adapted across databases. Searches will be updated before completion. Search results will be downloaded into EndNote, de-duplicated and imported into our review management system. The search strategy will be refined as needed. No date or language limits will be applied. Reference lists of included studies and relevant systematic reviews will be checked.

### Screening and selection

Two reviewers will independently screen titles and abstracts and then full texts. Disagreements will be resolved by discussion or by a third reviewer. Reasons for exclusions at full-text stage will be documented. Covidence software will be used.

### Data extraction

A standardised extraction form will be developed and piloted. Two reviewers will independently extract data. Disagreements will be resolved with a third reviewer. Authors will be contacted for missing data. Data will include: study characteristics (author, year, country, funding, conflicts of interest), participant details (age, gender, comorbidities, serving or ex-serving status, rank, branch and combat/non-combatant), intervention details (type, components, sessions, delivery format, therapist background, treatment duration, setting), comparator type and outcomes (including length of follow-up). Outcome data will be extracted for following timepoints: baseline, post-treatment and long-term follow-up. The data extracted will be reported in the characteristics of included studies tables, risk of bias tables and outcome data will be reported in forest plots and tables as appropriate.

For trials that randomised individuals, for each outcome, we will extract the number of patients randomised and the number of patients analysed for each study arm. For dichotomous outcomes, we will also extract the number of patients with the event for each study arm. For continuous outcomes, we will extract the mean and SD for each study group. For count outcomes, we will extract the number of events and the total person time at risk for each study arm. If this information is not reported, we will extract the summary statistics that are presented (eg, medians and ranges for each study arm).

For cluster-RCTs, we will extract the number of clusters randomised and number of clusters analysed per study arm as well as the number of patients randomised and analysed for each arm. We will also extract the average cluster size and the intra-cluster correlation coefficient. When trials adjusted the analyses for clustering, we will extract the cluster-adjusted measure of treatment effect and its variance. However, we will extract the same data as for trials that randomise individuals when trials did not adjust for clustering.

### Dismantling interventions

All intervention components will be classified independently by two reviewers using prespecified operational definitions and explicit coding rules, with disagreements resolved through discussion and, where necessary, consultation with a third reviewer. Equity-relevant characteristics will be extracted using the PROGRESS-Plus framework.^21^ We will develop a prespecified component dictionary to dismantle eligible interventions into constituent therapeutic ingredients, with coding undertaken at progressively finer levels of granularity (online supplemental table 2).

At level 1 (intervention family components), each study arm will be categorised into broad intervention families, including trauma-focused CBT, EMDR, non-trauma-focused CBT, mindfulness-based interventions, interpersonal psychotherapy, supportive therapy, psychodynamic therapy, exposure-based therapy, psychosocial rehabilitation-oriented interventions, other psychosocial interventions and comparator families such as waitlist, treatment as usual and active or attention control conditions. These level 1 categories will form the basis of the primary pairwise meta-analysis and component-NMA comparing intervention families

At level 2 (therapeutic and psychosocial components), level 1 intervention families will be further decomposed into shared therapeutic and psychosocial components, and separate component networks will be fitted within major intervention families where the evidence base is sufficient. These components will include, where relevant, psychoeducation, support, relaxation, affect regulation, cognitive restructuring, imaginal exposure, in vivo exposure, virtual reality exposure, mindfulness, phase-based or stabilisation elements, interpersonal focus, problem solving, behavioural activation, trauma processing or meaning making, homework, peer support, family or conjoint elements, case management, psychosocial rehabilitation, vocational rehabilitation, animal-assisted intervention and non-specific attention or placebo elements. Component-NMA will provide comparisons of the level 2 components and estimation of the contribution of shared ingredients within conceptually coherent treatment families. Separate component-NMAs will be performed within each intervention family, to avoid assuming that the same component operates equivalently across all intervention families.

At level 3 (procedural element components), selected level 2 components will be further dismantled into finer procedural elements. Examples include trauma narrative, repeated memory recounting, exposure hierarchies, breathing retraining, progressive muscle relaxation, grounding, cognitive coping, self-blame or guilt reappraisal, safety or trust belief modification, meditation practice, acceptance or decentring, social skills training, supported employment, community reintegration and animal-assisted therapy subtypes. For EMDR, modality-specific dismantling will be undertaken separately. At the first EMDR-specific level, we will code core procedural elements including trauma memory activation or retrieval, bilateral stimulation, dual attention or external attentional focus, negative cognition identification, positive cognition installation and somatic processing or body scan. Where trial reporting permits, more granular EMDR procedural variants, such as eye movements, alternating taps, alternating tones and repeated subjective distress monitoring, will be extracted for exploratory analyses.

Across all levels, components will be coded as present, absent or unclear using information from trial reports, supplementary files, manuals and protocol papers and will only be coded as present when explicitly described as intended therapeutic elements rather than inferred from intervention labels alone.

### Risk of bias and certainty of findings

Two reviewers will independently assess risk of bias for each included RCT using the Cochrane Risk of Bias 2 tool. Disagreements will be resolved with a third reviewer. Risk of bias assessments will be incorporated into the interpretation of findings and, where feasible, explored through sensitivity analyses excluding studies at high risk of bias or through meta-regression. We will also apply the Confidence in Network Meta-Analysis (CINeMA) framework^24^ to assess confidence in the results of the network meta-analysis.

### Data synthesis: general methods

Models will be run in R V.4.4.0 and WinBUGS V.1.4.3 with the R2WinBUGS package. A Bayesian framework with non-informative priors will be applied. Three chains will be run and assessed for convergence with Gelman Rubin statistics. Dichotomous outcomes will be presented as ORs; continuous outcomes as mean differences or standardised mean differences; and count outcomes as rate ratios. For each outcome, for each comparison, the posterior median measure of effect and its 95% credibility interval will be reported. We will carry out available case analyses, such that the analysis is based on the available data. We will also carry out sensitivity analyses based on risk of bias components. We will produce a network diagram for each outcome.

### Data synthesis: pairwise and network meta-analysis comparing interventions

We will produce network diagrams for each outcome. Pairwise and network meta-analyses will be carried out. Treatment rankings will be presented.^25^ Heterogeneity will be assessed through inspection of forest plots, and comparisons of effectiveness results and residual deviance and deviance information criterion of the fixed-effect and random-effects models. Consistency will be assessed through comparison of potential effect modifying characteristics (ie, age, gender and status of service), node-splitting and inconsistency models.^26^ Meta-regression may be applied if heterogeneity or inconsistency is detected (ie, average age; gender; status of service).^27^ Small-study effects will be assessed with funnel plots if data permit.

We will construct a network in which interventions will be grouped into major categories based on their theoretical underpinning, enabling comparisons across broader intervention classes rather than individual interventions.^28^

### Data synthesis: component network meta-analysis comparing components

Three levels of meta-analysis will be carried out. The following methods will be applied for level 1 analysis (intervention family components), level 2 analysis (therapeutic and psychosocial components) and level 3 analysis (procedural element components).

Component-NMA will be conducted that assumes that each component has a separate effect.^28 29^ Fixed-effect and random-effects models will be applied. The additive component-NMA model will be applied which makes an ‘additivity assumption’ that the component effects are independent, in other words, the total effectiveness of an intervention is equal to the sum of the effectiveness of its component parts. The validity of the additivity assumption will be assessed by (1) comparing the model fit and deviance information criterion and between trial variance of the NMA model with the additive component-NMA model; (2) evaluating whether each component’s effect differs across study designs (eg, is the effect of component A the same in studies of A vs C, and studies of A+B vs B+C).

If the additivity assumption is questionable, we will apply an interaction component-NMA model which is an extension of the additive model, and assumes that components interact with each other, such that the effectiveness of the intervention is either higher (synergistic) or lower (antagonistic) than the sum of the effectiveness of its component parts. Interactions will be predefined, before carrying out any analyses, based on their clinical plausibility determined by co-authors and evidence; and identifiability, that is the interacting components must have formed part of an included multi-component intervention. A forward selection approach will be used; first including each identifiable, clinically plausible interaction in separate additive models. Model fit, DIC and between trial variance of each interaction model will be compared with the NMA model. Additional interactions will be included in models until no gain in model fit is observed. If a high-order interaction is included (ie, three-way interaction or above), all relevant lower-order interactions will be included.

### Narrative synthesis

Where data cannot be pooled, narrative synthesis will be used. Where data were not available to be analysed using meta‐analysis, we will synthesise results narratively using Synthesis Without Meta‐analysis guidance.^30^ The narrative synthesis will be structured around the type of intervention, target population characteristics and outcome measures will be developed. Data will be presented as text, tables and diagrams.

### Cost-effectiveness

We will also include a narrative summary of any cost-effectiveness studies identified during the searches. Although this evidence is expected to be sparse, we will summarise reported economic outcomes such as intervention and healthcare costs, healthcare utilisation, cost savings, incremental cost-effectiveness ratios and quality-of-life measures including Quality Adjusted Life Years (QALYs). Where reported, we will also extract information on productivity, functioning and wider societal costs.

### Gaps and limitations

Given that we anticipate the quality of effectiveness studies for meta-analyses may be compromised by heterogeneity in study designs, intervention types and comparator groups, and that our preliminary searches for the cost-effectiveness review indicated a sparse evidence base, particular attention will be paid to identifying gaps, inconsistencies and methodological limitations. These include high risk of bias, inadequate reporting, limited generalisability across various military ranks or to non-military populations, small sample sizes, narrow time horizons, and insufficient reporting of costs or utility measures. By identifying these shortcomings, we aim to highlight areas requiring further research, such as the need for robust, large-scale economic evaluations that reflect the realities of providing psychological interventions to diverse military populations.

### Patient and public involvement

To ensure our findings are grounded in real-life experiences and deliver meaningful impact on practice and policy,^31^ the protocol has been prepared in collaboration with experts by lived experience (AB, DS). During the course of the review, we will establish a PPI group composed of <12 members, including serving and ex-serving military personnel, family members, academics, researchers and mental health professionals. This number will help us achieve diversity and inclusivity. Their involvement will be integral at every stage of the project, from search refinement to result interpretation and co-production of accessible summaries. PPI group will also guide the dissemination strategy to ensure that our work remains relevant and beneficial to those most impacted.

## Discussion

This review will provide the first comprehensive synthesis and (component) network meta-analysis focused solely on psychological interventions for PTSD in both serving and ex-serving military personnel. By using these advanced statistical methods, we will be able to compare the relative effectiveness of the different interventions as well as their components. This will allow us to go beyond simple head-to-head comparisons and produce a ranking of interventions and ultimately identify which therapeutic approaches are most effective for these vulnerable groups.

Military personnel face a unique combination of risk factors for PTSD. Many carry preservice trauma, which interacts with service-related trauma exposure. Furthermore, mental health-related stigma within the military culture as well as structural barriers to seeking support can delay treatment or symptom management.^17 32^ These unique military factors mean that the findings produced from studies focusing on the general population may not be applicable to military personnel. Hence, this review aims to address this gap directly by focusing on both serving and ex-serving personnel.

Our findings will inform both clinical practice and service delivery. For clinicians, our review aims to clarify which interventions work best, which components add value and which groups may need tailored approaches. For service planners and policymakers, these findings will provide an evidence base for investment decisions, highlight areas where services can be improved and suggest how support networks such as charities can complement formal care. For researchers, our review will map out gaps in the evidence base, such as the lack of longer-term follow-up data, limited data with regards to military-specific details (eg, branch of service, deployment history), impact on personnel families and carers and the limited evidence on cost-effectiveness.

However, we need to highlight that we anticipate several limitations. For example, the quality of trials may vary, and we anticipate that several studies will be based on small sample sizes, report short follow-ups or have inconsistent outcomes. We also anticipate high heterogeneity which will make comparisons challenging; hence, we aim to use methods like random effects models and meta-regression to account for this. We also anticipate that there will be studies not reporting sufficient detail regarding study characteristics, participant details and intervention details and outcomes. Cost-effectiveness data are likely to be sparse and/or not tailored to military contexts. We aim to address these limitations transparently and also use tools such as CINeMA to assess certainty to provide a robust and balanced picture of the evidence.

Overall, this work will generate knowledge that is directly relevant to improving treatment for military personnel with PTSD. It will also highlight where future research is most needed, especially in conducting robust economic evaluations, addressing equity, and developing interventions tailored to the specific experiences of military personnel.

## Ethics and dissemination

This research will establish comprehensive evidence about the effectiveness (and cost-effectiveness based on the available evidence) of psychological interventions for PTSD among military personnel. Some of the deliverables will be: (1) a full scientific report detailing methods, results, and conclusions, (2) one peer-reviewed publication focusing on effectiveness targeted for high-impact, open-access journals such as The Lancet and BMJ, (3) a consolidated and anonymised dataset of all trials of psychological interventions for PTSD in military personnel that will be a resource for future research and (4) evidence-based frameworks and strategic roadmaps to inform research and policy priorities, tailored to military contexts.

Stakeholders will be engaged through tailored communication strategies. Presentations will be made at conferences hosted by organisations such as the King’s Centre for Military Health Research and other high-profile military forums. Brief summaries, co-developed with our PPI coapplicants and members, will be disseminated via blogs, infographics, videos, and social media (eg, Twitter) to ensure the information is accessible to a broad audience. We will use platforms like Kudos and create a distinct project identity through the handle @Forces4Recovery on Twitter and related platforms, complementing question-and-answer sessions, workshops and direct engagement with patient and carer groups. This approach will maximise outreach and enable interactive discussions on project findings. Furthermore, our PPI co-applicants (ABac, AB and DS) have extensive connections to charities, single-service units and local government organisations. To support the formation of our PPI, we have secured and attached letters of support from charities such as Help For Heroes/Hidden Wounds and Walking with the Wounded, as well as NHS Trusts involved in the treatment of ex-serving military personnel with PTSD (Operation Restore). We anticipate that these organisations and Trusts will be instrumental in helping us recruit the 12 key stakeholders needed to guide the project’s key stages, ensuring strong engagement and impact across diverse communities.

The outputs will be directly integrated into healthcare systems through: presentation of findings in structured formats, such as CINeMA^24^ and PROGRESS+,^21^ to ensure usability for guideline developers, including NICE and the British Psychological Society; development of strategic frameworks to inform service delivery models in the National Health Services (NHS) and military healthcare; engagement with policymakers and military charities, such as Help for Heroes, to promote implementation across civilian and military settings; and dissemination to healthcare providers and service users through accessible materials and training to encourage practical adoption.

To support the findings of this important project, we will seek further funding to (1) develop and test the effectiveness, feasibility, and acceptability of an intervention based on the findings of our NMA project to further support military personnel with PTSD, (2) scale interventions and implement findings across diverse military branches and healthcare systems, (3) undertake larger-scale trials for the further refinement of effective therapeutic components, (4) investigate personalised PTSD treatment pathways and (5) develop digital tools and training programmes to embed research findings into routine practice. Funders may include National Institute for Health and Care Research, government departments, military charities and industry partnerships.

## Supplementary material

10.1136/bmjopen-2026-124637 online supplemental table 1

10.1136/bmjopen-2026-124637 online supplemental table 2
