# Supplementary material for: Psychosocial interventions for military personnel with PTSD: protocol of a systematic review and network meta-analysis
Source: BMJ Open. 2026 Oct 5;16(10):e124637. doi: 10.1136/bmjopen-2026-124637 (PMC13647000; doi:10.1136/bmjopen-2026-124637)
Supplement: Supplementary file 2 [file bmjopen-16-10-s001.docx]

Supplementary Table 1: Search strategy adapted for PubMed

| Population | ("Military Personnel"[Mesh] OR "Veterans"[Mesh] OR veteran*[Title/Abstract] OR veterans[Title/Abstract] OR "ex-service"[Title/Abstract] OR "ex servic*"[Title/Abstract] OR "former service*"[Title/Abstract] OR military[Title/Abstract] OR militar*[Title/Abstract] OR "armed force*"[Title/Abstract] OR "armed service*"[Title/Abstract] OR "service member*"[Title/Abstract] OR servicemember*[Title/Abstract] OR "service personnel"[Title/Abstract] OR soldier*[Title/Abstract] OR troop*[Title/Abstract] OR army[Title/Abstract] OR navy[Title/Abstract] OR naval[Title/Abstract] OR "air force"[Title/Abstract] OR airforce[Title/Abstract] OR airman[Title/Abstract] OR airmen[Title/Abstract] OR marine*[Title/Abstract] OR "coast guard"[Title/Abstract] OR reservist*[Title/Abstract] OR "reserve force*"[Title/Abstract] OR "national guard"[Title/Abstract] OR "active duty"[Title/Abstract] OR "active service"[Title/Abstract] OR "serving personnel"[Title/Abstract] OR "military officer*"[Title/Abstract] OR "military staff"[Title/Abstract] OR infantry[Title/Abstract] OR combatant*[Title/Abstract] OR warfighter*[Title/Abstract] OR "special force*"[Title/Abstract] OR "special operations"[Title/Abstract]) |
| --- | --- |
| Outcome | ("Stress Disorders, Post-Traumatic"[Mesh] OR PTSD[Title/Abstract] OR "post-traumatic stress disorder"[Title/Abstract] OR "post traumatic stress disorder"[Title/Abstract] OR "post-traumatic stress symptoms"[Title/Abstract] OR "post traumatic stress symptoms"[Title/Abstract] OR PTSS[Title/Abstract] OR "combat stress"[Title/Abstract] OR "traumatic stress"[Title/Abstract]) |
| Interventions | ("cognitive therapy"[Title/Abstract] OR "cognitive therapies"[Title/Abstract] OR "cognitive behavioural therapy"[Title/Abstract] OR "cognitive behavioral therapy"[Title/Abstract] OR "cognitive behavioural treatment*"[Title/Abstract] OR "cognitive behavioral treatment*"[Title/Abstract] OR "cognitive restructuring"[Title/Abstract] OR "cognitive restructuring therapy"[Title/Abstract] OR "cognitive processing therapy"[Title/Abstract] OR "CBT"[Title/Abstract] OR "TF-CBT"[Title/Abstract] OR "TFCBT"[Title/Abstract] OR "trauma-focused CBT"[Title/Abstract] OR "trauma focused CBT"[Title/Abstract] OR "trauma-focused cognitive behavioural therapy"[Title/Abstract] OR "trauma-focused cognitive behavioral therapy"[Title/Abstract] OR "cognitive trauma therapy"[Title/Abstract] OR "behavior therapy"[Title/Abstract] OR "behaviour therapy"[Title/Abstract] OR "behavioral therapy"[Title/Abstract] OR "behavioural therapy"[Title/Abstract] OR "behavior modification"[Title/Abstract] OR "behaviour modification"[Title/Abstract] OR "dialectical behavior therapy"[Title/Abstract] OR "dialectical behaviour therapy"[Title/Abstract] OR "DBT"[Title/Abstract] OR "acceptance and commitment therapy"[Title/Abstract] OR "ACT"[Title/Abstract] OR "mindfulness"[Title/Abstract] OR "mindfulness-based cognitive therapy"[Title/Abstract] OR "MBCT"[Title/Abstract] OR "mindfulness-based stress reduction"[Title/Abstract] OR "MBSR"[Title/Abstract] OR "biofeedback"[Title/Abstract] OR "neurofeedback"[Title/Abstract] OR "sensory feedback"[Title/Abstract] OR "eye movement desensitization and reprocessing"[Title/Abstract] OR "eye movement desensitisation and reprocessing"[Title/Abstract] OR "eye movement desensitization reprocessing"[Title/Abstract] OR "eye movement desensitisation reprocessing"[Title/Abstract] OR "EMDR"[Title/Abstract] OR "exposure therapy"[Title/Abstract] OR "exposure therapies"[Title/Abstract] OR "exposure treatment*"[Title/Abstract] OR "prolonged exposure"[Title/Abstract] OR "prolonged exposure therapy"[Title/Abstract] OR "imaginal exposure"[Title/Abstract] OR "in vivo exposure"[Title/Abstract] OR "live exposure"[Title/Abstract] OR "virtual reality exposure therapy"[Title/Abstract] OR "VRET"[Title/Abstract] OR "implosive therapy"[Title/Abstract] OR "flooding"[Title/Abstract] OR "stress inoculation training"[Title/Abstract] OR "stress management"[Title/Abstract] OR "relaxation training"[Title/Abstract] OR "relaxation therapy"[Title/Abstract] OR "hypnosis"[Title/Abstract] OR "hypnotherapy"[Title/Abstract] OR "psychotherapy"[Title/Abstract] OR "psychotherapies"[Title/Abstract] OR "brief psychotherapy"[Title/Abstract] OR "group psychotherapy"[Title/Abstract] OR "psychodynamic therapy"[Title/Abstract] OR "interpersonal psychotherapy"[Title/Abstract] OR "IPT"[Title/Abstract] OR "supportive therapy"[Title/Abstract] OR "counseling"[Title/Abstract] OR "counselling"[Title/Abstract] OR "non-directive counseling"[Title/Abstract] OR "nondirective counseling"[Title/Abstract] OR "non-directive counselling"[Title/Abstract] OR "nondirective counselling"[Title/Abstract] OR "compassion-focused therapy"[Title/Abstract] OR "compassion focused therapy"[Title/Abstract] OR "compassion therapy"[Title/Abstract] OR "schema therapy"[Title/Abstract] OR "metacognitive therapy"[Title/Abstract] OR "accelerated resolution therapy"[Title/Abstract] OR "sensorimotor therapy"[Title/Abstract] OR "psychoeducation"[Title/Abstract] OR "psychoeducational intervention*"[Title/Abstract] OR "psychosocial intervention*"[Title/Abstract] OR "psychosocial treatment*"[Title/Abstract] OR "psychosocial therap*"[Title/Abstract] OR "psychosocial support"[Title/Abstract] OR "psychosocial rehabilitation"[Title/Abstract] OR "psychiatric rehabilitation"[Title/Abstract] OR "vocational rehabilitation"[Title/Abstract] OR "supported employment"[Title/Abstract] OR "occupational rehabilitation"[Title/Abstract] OR "occupational therapy"[Title/Abstract] OR "peer support"[Title/Abstract] OR "peer-delivered intervention*"[Title/Abstract] OR "peer led intervention*"[Title/Abstract] OR "peer-led intervention*"[Title/Abstract] OR "group intervention*"[Title/Abstract] OR "family intervention*"[Title/Abstract] OR "family therap*"[Title/Abstract] OR "skills training"[Title/Abstract] OR "social skills training"[Title/Abstract] OR "coping skills training"[Title/Abstract] OR "problem solving"[Title/Abstract] OR "problem-solving"[Title/Abstract] OR "problem solving therapy"[Title/Abstract] OR "problem-solving therapy"[Title/Abstract] OR "self-management"[Title/Abstract] OR "self management"[Title/Abstract] OR "case management"[Title/Abstract] OR "supportive counseling"[Title/Abstract] OR "supportive counselling"[Title/Abstract] OR "animal-assisted therapy"[Title/Abstract] OR "animal assisted therapy"[Title/Abstract] OR "animal-assisted intervention*"[Title/Abstract] OR "animal assisted intervention*"[Title/Abstract] OR "equine-assisted therapy"[Title/Abstract] OR "equine assisted therapy"[Title/Abstract] OR "canine-assisted therapy"[Title/Abstract] OR "canine assisted therapy"[Title/Abstract] OR "dog-assisted therapy"[Title/Abstract] OR "dog assisted therapy"[Title/Abstract] OR "rehabilitation"[Title/Abstract] OR "recovery-oriented intervention*"[Title/Abstract] OR "community reintegration"[Title/Abstract]) |
| Design | ("randomized controlled trial"[Title/Abstract] OR "randomised controlled trial"[Title/Abstract] OR "randomized trial"[Title/Abstract] OR "randomised trial"[Title/Abstract] OR "randomized clinical trial"[Title/Abstract] OR "randomised clinical trial"[Title/Abstract] OR "clinical trial"[Title/Abstract] OR "controlled trial"[Title/Abstract] OR "pragmatic trial"[Title/Abstract] OR "effectiveness trial"[Title/Abstract] OR "efficacy trial"[Title/Abstract] OR "cluster randomized trial"[Title/Abstract] OR "cluster randomised trial"[Title/Abstract] OR "cluster randomized controlled trial"[Title/Abstract] OR "cluster randomised controlled trial"[Title/Abstract] OR "cluster trial"[Title/Abstract] OR "cluster controlled trial"[Title/Abstract] OR "group randomized trial"[Title/Abstract] OR "group randomised trial"[Title/Abstract] OR "group randomized controlled trial"[Title/Abstract] OR "stepped wedge trial"[Title/Abstract] OR "stepped-wedge trial"[Title/Abstract] OR "pilot trial"[Title/Abstract] OR "feasibility trial"[Title/Abstract] OR "pilot randomized trial"[Title/Abstract] OR "pilot randomised trial"[Title/Abstract] OR "pilot randomized controlled trial"[Title/Abstract] OR "pilot randomised controlled trial"[Title/Abstract] OR "effectiveness"[Title/Abstract] OR "treatment effectiveness"[Title/Abstract] OR "intervention effectiveness"[Title/Abstract] OR "comparative effectiveness"[Title/Abstract] OR "cost effectiveness"[Title/Abstract] OR "cost-effectiveness"[Title/Abstract] OR "cost utility"[Title/Abstract] OR "cost-utility"[Title/Abstract] OR "cost benefit"[Title/Abstract] OR "cost-benefit"[Title/Abstract] OR "economic evaluation"[Title/Abstract] OR "health economic evaluation"[Title/Abstract] OR "cost analysis"[Title/Abstract] OR "cost-analysis"[Title/Abstract] OR "cost minimization"[Title/Abstract] OR "cost-minimization"[Title/Abstract] OR "cost consequence"[Title/Abstract] OR "cost-consequence"[Title/Abstract] OR "incremental cost effectiveness ratio"[Title/Abstract] OR "incremental cost-effectiveness ratio"[Title/Abstract] OR "ICER"[Title/Abstract] OR "quality-adjusted life year"[Title/Abstract] OR "quality-adjusted life years"[Title/Abstract] OR "QALY"[Title/Abstract] OR "QALYs"[Title/Abstract]) |
