# Supplementary material for: Psychosocial interventions for military personnel with PTSD: protocol of a systematic review and network meta-analysis
Source: BMJ Open. 2026 Oct 5;16(10):e124637. doi: 10.1136/bmjopen-2026-124637 (PMC13647000; doi:10.1136/bmjopen-2026-124637)
Supplement: Supplementary file 3 [file bmjopen-16-10-s002.docx]

Supplementary Table 2: Hierarchical component dictionary for coding psychological and psychosocial interventions for PTSD

| **Component** | **Operational definition** |
| --- | --- |
| **Level 1: Intervention categories** | |
| **Trauma-focused CBT (TF-CBT)** | A CBT-based intervention explicitly focused on processing traumatic memories and/or trauma-related cognitions, typically combining exposure-based and/or cognitive techniques. |
| **EMDR** | Eye Movement Desensitization and Reprocessing or related protocols involving trauma memory activation with bilateral stimulation. |
| **Non-trauma-focused CBT** | A CBT-based intervention that does not directly focus on trauma memory processing, but instead targets general symptoms, coping, mood, anxiety, or functioning. |
| **Mindfulness-based intervention** | An intervention in which mindfulness, meditation, acceptance, or present-moment awareness is the principal therapeutic model. |
| **Interpersonal psychotherapy (IPT)** | A psychotherapy focused on interpersonal relationships, role transitions, interpersonal disputes, grief, and social functioning. |
| **Supportive therapy / counselling** | A primarily non-directive, supportive, empathic, or client-centred intervention without structured trauma-focused or CBT-specific therapeutic procedures. |
| **Psychodynamic therapy** | A therapy based on psychodynamic principles, including unconscious processes, relational patterns, conflict, or developmental formulations. |
| **Exposure-based therapy** | A treatment primarily based on imaginal, in vivo, or other forms of exposure to trauma memories or reminders. |
| **Psychosocial rehabilitation / rehabilitation-oriented intervention** | A structured intervention focused on recovery, functioning, social reintegration, occupational functioning, or community participation. |
| **Other psychosocial intervention** | Any eligible psychosocial or psychological intervention not captured by the above categories. |
| **Waitlist** | Participants receive no active intervention during the study period but may receive treatment later. |
| **Treatment as usual (TAU)** | Participants continue receiving routine or standard care without a structured study intervention. |
| **Active control / attention control** | A comparator designed to control for attention, expectancy, therapist contact, or non-specific treatment effects. |
| **Delivery** | Digital/therapist/task-shared |
| **Delivery format** | Self-guided/Individual/group |
| **Level 2 dismantling** | |
| **Psychoeducation** | Structured information about trauma, PTSD symptoms, common post-traumatic reactions, treatment rationale, or recovery processes. |
| **Support** | Non-directive emotional support, empathic listening, validation, encouragement, or supportive discussion. |
| **Relaxation** | Techniques intended to reduce physiological arousal or tension. |
| **Affect regulation / emotion regulation** | Strategies teaching recognition, modulation, tolerance, or management of intense emotions. |
| **Cognitive restructuring** | Identification, evaluation, and modification of maladaptive thoughts, appraisals, beliefs, or interpretations related to trauma. |
| **Imaginal exposure** | Deliberate therapeutic revisiting of the trauma memory through narration, reliving, recounting, or detailed imagery. |
| **In vivo exposure** | Graduated confrontation with objectively safe trauma reminders, situations, places, people, or activities that are being avoided. |
| **Virtual reality exposure** | Exposure to trauma-related cues or scenarios delivered through virtual reality or immersive simulation. |
| **Mindfulness** | Training in present-moment awareness, non-judgemental attention, acceptance, meditation, or mindful observation of thoughts and feelings. |
| **Phase-based / stabilisation** | An explicitly phased intervention structure in which an initial stabilisation, safety, or skills-building phase precedes trauma processing. |
| **Interpersonal focus** | Techniques targeting interpersonal disputes, grief, role transitions, social functioning, communication, or relationship patterns. |
| **Problem solving** | Structured identification of practical problems, generation of solutions, decision-making, and action planning. |
| **Behavioural activation / activity scheduling** | Techniques aimed at increasing meaningful, rewarding, or routine activities and reducing withdrawal or avoidance. |
| **Trauma processing / meaning making** | Therapeutic work aimed at integrating, reinterpreting, or making sense of the traumatic experience and its meaning. |
| **Homework / between-session practice** | Structured practice tasks assigned between sessions to consolidate therapeutic skills or exposure exercises. |
| **Peer support / peer delivery** | Intervention content delivered by peers or incorporating structured peer support as a central ingredient. |
| **Family / conjoint component** | Involvement of family members, partners, caregivers, or conjoint sessions as part of treatment. |
| **Case management / care coordination** | Practical support, service navigation, linkage with services, monitoring, or coordination across sectors. |
| **Psychosocial rehabilitation** | Structured support focused on recovery, community reintegration, social skills, independent living, or functional improvement. |
| **Vocational rehabilitation** | Intervention targeting return to work, supported employment, occupational functioning, or work reintegration. |
| **Animal-assisted intervention** | Therapeutic involvement of animals, such as equine-assisted or canine-assisted therapy, as a structured treatment element. |
| **Attention / placebo component** | Receipt of therapist attention, session time, credible therapeutic contact, or structured non-specific treatment input. |
| **Attachment-Focused Work** | Addressing trauma-related attachment patterns, trust, and relational safety. |
| **Therapeutic Alliance Enhancement** | Explicit strategies to strengthen collaboration, trust, and engagement. |
| **Group Process** | Use of peer interaction, shared experiences, and group dynamics as therapeutic mechanisms. |
| **Level 3 dismantling: Exploratory analysis** | |
| **Trauma narrative** | Development or retelling of a structured narrative of the traumatic experience. |
| **Reliving / repeated memory recounting** | Repeated revisiting of the traumatic memory in detail, often session-by-session. |
| **Exposure hierarchy** | A planned hierarchy of feared or avoided trauma reminders used to structure exposure work. |
| **Breathing retraining** | Training in controlled breathing to reduce distress or hyperarousal. |
| **Progressive muscle relaxation** | Sequential tensing and relaxing of muscle groups to reduce physiological tension. |
| **Grounding** | Techniques used to reorient attention to the present and reduce dissociation or acute distress. |
| **Cognitive coping** | Teaching patients to identify links between thoughts, feelings, and behaviours and respond more adaptively. |
| **Self-blame / guilt reappraisal** | Specific cognitive work focused on reducing guilt, shame, or self-blame. |
| **Safety / trust belief modification** | Cognitive work targeting beliefs about safety, trust, control, or vulnerability. |
| **Meditation practice** | Formal seated, guided, or repeated mindfulness meditation exercises. |
| **Acceptance / decentering** | Techniques promoting acceptance of internal experiences or distancing from thoughts. |
| **Social skills training** | Structured training to improve communication, assertiveness, or interpersonal functioning. |
| **Supported employment** | Employment support embedded within vocational rehabilitation. |
| **Community reintegration** | Intervention aimed at restoring community participation, role functioning, or social reintegration. |
| **Equine-assisted therapy** | Therapeutic intervention involving horses. |
| **Canine-assisted therapy** | Therapeutic intervention involving dogs. |
| **Level 2: EMDR only** | |
| **Trauma memory activation / retrieval** | Deliberate activation of the traumatic memory, target image, associated affect, bodily sensations, and maladaptive cognition during treatment. |
| **Bilateral stimulation** | Alternating bilateral sensory stimulation delivered during trauma processing. |
| **Dual attention / external attentional focus** | Simultaneous engagement with the traumatic memory while attending to an external stimulus or task. |
| **Negative cognition identification** | Identification of a maladaptive trauma-linked belief about the self-associated with the target memory. |
| **Positive cognition installation** | Strengthening or “installing” a preferred adaptive belief linked to the target memory after desensitization. |
| **Somatic processing / body scan** | Assessment and processing of residual bodily disturbance linked to the traumatic memory. |
